# Leucine modulates dynamic phosphorylation events in insulin signaling pathway and enhances insulin-dependent glycogen synthesis in human skeletal muscle cells

**DOI:** 10.1186/1471-2121-15-9

**Published:** 2014-03-20

**Authors:** Barbara Di Camillo, Federica Eduati, Sreekumaran K Nair, Angelo Avogaro, Gianna M Toffolo

**Affiliations:** 1 Department of Information Engineering, University of Padua, Padua, Italy; 2 Endocrine Research Unit, Mayo Clinic, Rochester, MN, USA; 3 Department of Clinical and Experimental Medicine, University of Padua, Padua, Italy and Venetian Institute of Molecular Medicine (VIMM), Padova, Italy

## Abstract

**Background:**

Branched-chain amino acids, especially leucine, are known to interact with insulin signaling pathway and glucose metabolism. However, the mechanism by which this is exerted, remain to be clearly defined. In order to examine the effect of leucine on muscle insulin signaling, a set of experiments was carried out to quantitate phosphorylation events along the insulin signaling pathway in human skeletal muscle cell cultures. Cells were exposed to insulin, leucine or both, and phosphorylation events of key insulin signaling molecules were tracked over time so as to monitor time-related responses that characterize the signaling events and could be missed by a single sampling strategy limited to pre/post stimulus events.

**Results:**

Leucine is shown to increase the magnitude of insulin-dependent phosphorylation of protein kinase B (AKT) at Ser473 and glycogen synthase kinase (GSK3β) at Ser21-9. Glycogen synthesis follows the same pattern of GSK3β, with a significant increase at 100 μM leucine plus insulin stimulus. Moreover, data do not show any statistically significant increase of pGSK3β and glycogen synthesis at higher leucine concentrations. Leucine is also shown to increase the magnitude of insulin-mediated extracellularly regulated kinase (ERK) phosphorylation; however, differently from AKT and GSK3β, ERK shows a transient behavior, with an early peak response, followed by a return to the baseline condition.

**Conclusions:**

These experiments demonstrate a complementary effect of leucine on insulin signaling in a human skeletal muscle cell culture, promoting insulin-activated GSK3β phosphorylation and glycogen synthesis.

## Background

**Figure 1:**
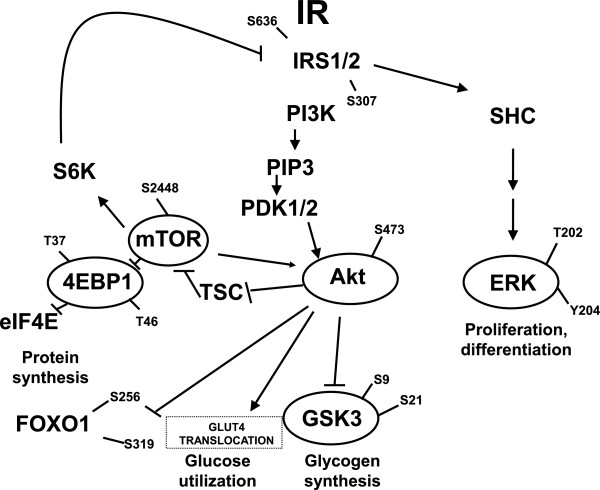
**Insulin signaling pathway.** The scheme represents the current knowledge on insulin signaling downstream of the insulin receptor (IR). Proteins that were measured by our experiment are circled and marked with the corresponding phosphorylation sites.

Insulin signaling pathway [1] is characterized by cascades of phosphorylation events, which activate and deactivate molecules critical for physiological responses such as glucose uptake, protein synthesis, and glycogen production (Figure 1). In particular, insulin interacts with the insulin receptor and modulates its signaling through phosphorylation of insulin receptor substrate 1 (IRS1) or its homolog IRS2. The C-terminal tail of IRS1 and IRS2, which contains several tyrosine and serine phosphorylation sites, binds the enzyme phosphoinositide 3-kinase (PI3K). PI3K converts PI(4,5)P2, a membrane inositol phospholipid, into PI(3,4,5)P3, which recruits 3-phosphoinositide-dependent protein kinase-1 (PDK1) to the plasma membrane, where PDK1 activates protein kinase B (AKT). AKT phosphorylates many substrates important for cell survival, regulation of growth and glycogen synthesis, gene expression, and glucose uptake. In particular, AKT acts on glycogen synthase kinase GSK3β by phosphorylating and thus inactivating it and, in turn, activating the enzyme glycogen synthase which is responsible for adding UDP-glucose to a growing chain of glycogen. AKT also acts on the mammalian target of rapamycin (mTOR), a multi-domain Serine/Threonine kinase that phosphorylates eukaryotic initiation factor 4E binding protein 1 (4EBP1), which inactivates eIF4E, allowing initiation of translation. In addition to 4EBP1, mTOR also regulates S6 kinase (S6K), which, besides leading to an increase in protein synthesis and cell proliferation, exerts a feedback effect on IRS1. A third target of AKT is forkhead homolog in rhabdomyosarcoma 1 (FOXO1). The specific function of this transcription factor has not yet been well determined; however, it is known to act as a regulator of cell response to oxidative stress and may play a role in myogenic growth and differentiation.

Notably, the insulin signaling network involves at least another major pathway, the extracellularly regulated kinase (ERK) - mitogen activated protein kinase (MAPK). The ERK-MAPK pathway can be considered as a general signaling pathway that is activated by a number of growth factors, including insulin itself, all leading to enhanced cell growth. Insulin triggers the ERK-MAPK pathway upon binding of Grb2 (growth factor receptor binding protein 2) to Tyr-phosphorylated Shc or IRS proteins via its SH2 (Src-homology-2) domain. The signal is then propagated to ERK1 and ERK2, which are translocated to the nucleus where they activate cell proliferation.

Branched-chain amino acids (BCAAs), especially leucine, are known to interact with insulin signaling pathway. In particular, leucine is an essential amino acid, which has been shown to promote protein synthesis [2,3]; however, the mechanism by which this is exerted is still controversial and seems to depend on cell type. An increase of the phosphorylation state of mTOR and its downstream regulators of protein synthesis S6K and 4EBP1 has been observed in mouse and rat muscle cells treated with leucine plus insulin [4,5]. On the opposite, in Zeanandin et al. [6], mTOR/S6K signaling pathway was not significantly altered in muscle of rats subjected to long-term dietary leucine excess, whereas it was increased in rat adipose tissue.

Leucine was also reported to exert inhibition on the early steps of insulin signaling leading to insulin resistance in rats after 5 weeks of dietary leucine supplementation [7]. Moreover, leucine deprivation in human HepG2 cells and mouse primary hepatocytes has been shown to improve hepatic insulin sensitivity by decreasing mammalian target of rapamycin/S6K1 signaling [8]. In contrast with the above findings, a high-protein diet was found to lower postprandial blood glucose in persons with type 2 diabetes and improve overall glucose control [9]. Along this line, Macotela et al. have recently reported, using rodent models, that doubling dietary leucine for eight weeks can reverse many of the metabolic abnormalities associated with high fat diet-induced obesity, improving glucose tolerance [10].

Since many events secondary to leucine administration can occur during in vivo studies, but not during in vitro experiments, there is still quite a bit of uncertainty on the precise role of leucine in regulating glucose metabolism. Perhaps leucine also acts at multiple points along the insulin signaling pathway and its overall effect may be the combination of its multiple effects; thus, examining the effects of leucine on mTOR targets as well as classical insulin signaling molecules such as AKT and ERK may be relevant for elucidating its effects.

The aim of the current study is to examine the effect of leucine on insulin signaling cascade in a human skeletal muscle cell line. To accomplish this goal it is crucial to track phosphorylation events over time so to monitor time-related changes of signaling events. This also allows monitoring transient changes in phosphorylation of signaling proteins, which may be missed when sample collection is limited to pre and post stimulus events. The only practical way to monitor dynamic response is to use a cell line, because it is not possible to perform multiple, rapid sampling experiments in animals. Moreover, an in vitro study offers the opportunity to assess the direct effect of leucine rather than the effects that occur through secondary events.

A skeletal muscle cell line was chosen to this purpose, because this tissue plays a key role in whole body glucose metabolism, by accounting for 70-80% of insulin-mediated glucose uptake in healthy humans [11,12]. Cell lines were exposed to insulin, leucine and leucine + insulin stimulus and total and phosphorylated AKT, ERK1/2, GSK3β, FOXO1, mTOR, 4EBP1, P70S6K were measured using Western blots at 6 time points collected in the 60 minutes following stimulation. In particular, to identify proteins whose phosphorylated/total ratio changes in time, we applied a statistical test specifically designed to select differentially expressed proteins based on their overall dynamic profile monitored during a perturbation. Glycogen synthesis at 90 minutes was also monitored in the three experimental conditions and at four different leucine concentrations in the leucine + insulin experiment.

## Results

**Figure 2:**
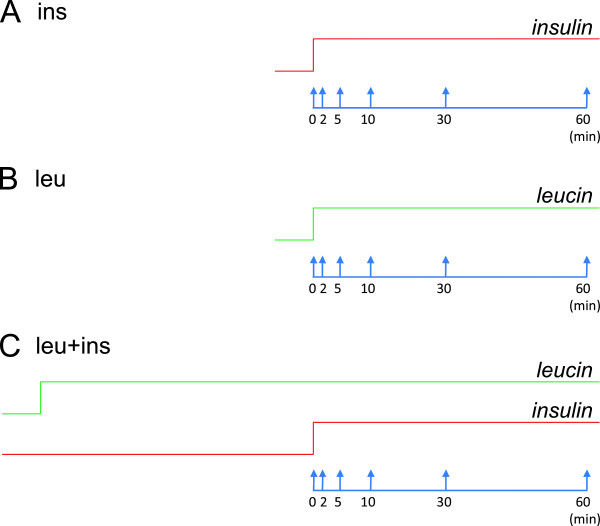
**Experimental setup.** Three experimental conditions: **A)** Stimulation with insulin at time 0′ (ins); **B)** Stimulation with leucine at time 0′ (leu); **C)** 60 minutes pre-incubation with leucine followed by stimulation with insulin at time 0′ (leu + ins).

Muscle cells were deprived of serum overnight prior to exposure to three different stimuli: A) cells were stimulated with insulin at time 0′; B) cells were stimulated with leucine at time 0′; C) cells were pre-incubated with leucine for one hour and then stimulated with insulin (Figure 2). Cells were exposed to stimuli for the following durations: 0′, 2′, 5′, 10′, 30′, and 60′ and AKT, pAKT-S473, ERK1/2, ppERK1/2-T202-Y204, GSK3β, ppGSK3β-S21-S9, FOXO1a, pFOXO1-S256, mTOR, pmTOR-S2448, 4EBP1, pp4EBP1-T37-T46, P70S6K, and pP70S6K-T389 were measured using Western blots. Each experiment was repeated three times including cell cultures, so to have three available biological replicates.

It is important to note that leucine does not alter insulin level, since average insulin concentration across the three experimental replicates is equal to 88 ± 13 nM when only the insulin stimulus is applied, and remains similar, i.e. equal to 87 ± 6 nM, when leucine is added to the medium (data expressed as mean ± standard deviation).

**Figure 3:**
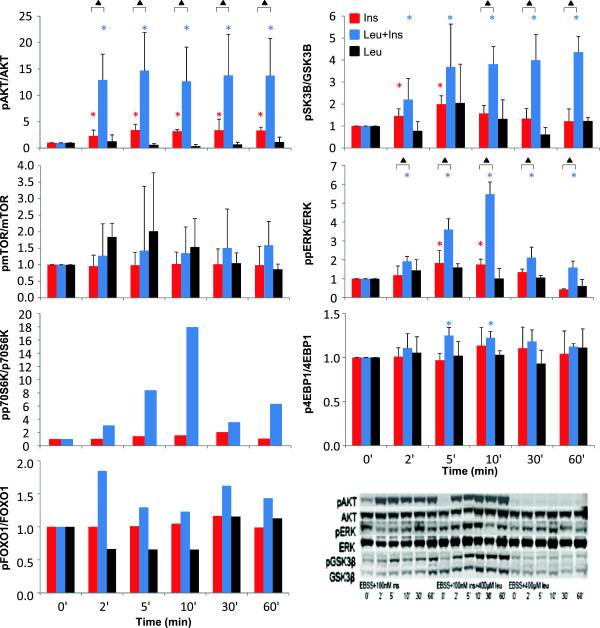
**Effects of insulin, leucine and leucine + insulin treatments on proteins’ phosphorylation.** Time-series expression profiles showing average and standard deviation of the data normalized to their time 0′ value and expressed as phosphorylated/total ratios under insulin (red), leucine (black) and leucine + insulin (blue) stimulus. Stars indicate significant p-values of t-tests performed on single time point for each treatment vs. the baseline. Triangles indicate significant p-values of t-tests performed on single time point for insulin vs. leucine + insulin treatment. Representative Western blots of total and phosphorylated AKT, GSK3β and ERK proteins are shown in the lowest right panel.

The average dynamic response of proteins treated with insulin, leucine and leucine + insulin is depicted in Figure 3. As explained in section Methods, data are expressed relative to the baseline band to correct for the antibody efficiency. Moreover, to allow for comparison among different membranes, the ratios between phosphorylated and total proteins is calculated. Figure 3 also shows standard deviations calculated across the three biological replicates and the results of the t-tests performed on single time point for each treatment vs. the baseline (stars indicate p-values lower than 0.05) and on single time point for insulin vs. leucine + insulin treatment comparisons (triangles indicate p-values lower than 0.05).

In addition to that, the “Bounded Area” method, originally described in [13], was applied to the time courses of the ratio between phosphorylated and total proteins monitored in a single replicate, to identify, for each protein, the replicates whose overall dynamic profile is significantly modulated by insulin, leucine and/or leucine + insulin treatment. The results are summarized in Table 1, in terms of False Discovery Rate corrected p-value (empty cells indicate that data were not available). For FOXO1 and P70S6K, for example, results refer to one replicate only, since in two replicates the phosphorylated protein concentration at time t = 0′ was below the detection threshold, thus making impossible to perform the normalization step to correct for antibody efficiency (see section Methods).

**Table 1:** Results of the bounded area test

**Treatment**	**INS**			**LEU**			**LEU + INS**			**LEU + INS vs. INS**		
**Biological replicate**	**R1**	**R2**	**R3**	**R1**	**R2**	**R3**	**R1**	**R2**	**R3**	**R1**	**R2**	**R3**
**pAKT/AKT**	7e^−3^	7e^−5^	4e^−3^	>0.5	>0.5	>0.5	3e^−5^	1e^−5^	2e^−5^	2e^−2^	1e^−5^	4e^−6^
**pGSK3β/GSK3β**	3e^−6^	2e^−2^	4e^−2^	>0.5	>0.5	>0.5	3e^−4^	9e^−4^	2e^−2^	3e^−2^	8e^−4^	1e^−3^
**pmTOR/mTOR**	>0.5	>0.5	>0.5	>0.5	3e^−2^	>0.5	>0.5	1e^−2^	2e^−4^	>0.5	>0.5	>0.5
**pERK/ERK**	>0.5	9e^−3^	4e^−2^	>0.5	>0.5	>0.5	2e^−2^	4e^−2^	2e^−2^	2e^−2^	4e^−2^	2e^−2^
**pp70S6K/p70S6K**		4e^−2^						3e^−2^			8e^−5^	
**p4EBP1/4EBP1**	>0.5	>0.5	>0.5	>0.5	>0.5	>0.5	6e^−4^	2e^−2^	2e^−2^	>0.5	>0.5	>0.5
**pFOXO1/FOXO1**			>0.5			>0.5			1e^−2^			>0.5

Results of the Bounded Area method [13], applied to the time courses of the ratio between phosphorylated and total proteins monitored in a single replicate, to identify, for each protein, the replicates whose overall dynamic profile is significantly modulated by insulin, leucine and/or leucine + insulin treatment. False Discovery Rate corrected p-values are reported. Empty cells indicate that data are not available.

According to our results, leucine alone has virtually no effect on monitored phosphorylation events, since variations with respect to baseline are never significant when performing a t-test on single time-points across the three replicates (Figure 3, black bars) and are significant only for one replicate of mTOR when the Bounded Area method is applied to consider the overall dynamic profile of each protein (Table 1).

Insulin alone has a major upregulatory effect on AKT, ERK and GSK3β (Figure 3, red bars indicating average measurements under insulin stimulus with red stars indicating statistical significance with respect to the baseline). In particular, pAKT/AKT shows an immediate significant rise at time 2′, which is maintained in the following 60′, whereas pERK/ERK and pGSK3β/GSK3β return to the baseline after an early rise. Considered on the overall dynamic profile of each protein (Table 1), insulin effect is significant in all biological replicates for AKT and GSK3β, and in two out of three replicates for ERK. Addition of leucine potentiates the insulin effect on phosphorylation (Figure 3, blue bars) since pAKT/AKT, pERK/ERK and pGSK3β/GSK3β are significantly higher than baseline at all sampling times (Figure 3, blu stars), and are significantly higher than with insulin alone at most sampling times (Figure 3, black triangles). In terms of overall time-series effect (Table 1), pAKT/AKT, pERK/ERK and pGSK3β/GSK3β are all upregulated in all biological replicates with leucine + insulin both with respect to baseline and in comparison with insulin alone.

The pattern of pmTOR/mTOR, which is responsible for 4EBP1 and p70S6K phosphorylation, is more difficult to be deciphered given the high variability among the three replicates. Single time point comparisons (Figure 3) do not allow to detect any significant difference after insulin stimulation, both alone and in combination with leucine, whereas the overall profile (Table 1) is not significantly expressed in any replicate during insulin stimulus, but indicates up-regulation in two out of three replicates under leucine + insulin stimulus.

p4EBP1/4EBP1 appears to be significantly up-regulated only at time 5′ and 10′ following leucine + insulin stimulus (Figure 3), but this is sufficient to elicit a significant overall effect of leucine + insulin in all replicates (Table 1), when compared to baseline.

As regards FOXO1, which is a target of AKT, data shown in Figure 3 suggest some effect under leucine + insulin stimulus, but since just one replicate is available, it is not possible to derive any statistical conclusion. Application of the Bounded Area method to this single replicate, however, confirms an up-phosphorylation under leucine + insulin stimulus.

Even for pp70S6K/p70S6K only one replicate is available. The time course data (Figure 3) suggest a 2-fold up-regulation by insulin, potentiated by leucine to 18-fold up-regulation. The overall effect on the available replicates is significant with insulin as well as with leucine + insulin and is still significant when comparing leucine + insulin vs. insulin stimulus.

**Figure 4:**
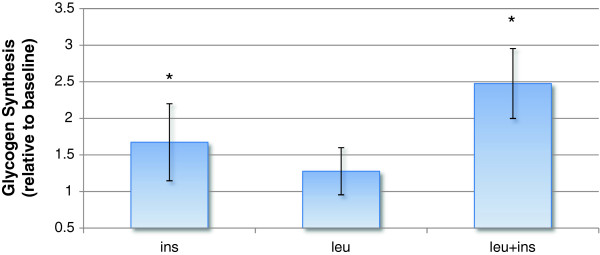
**Effects of insulin and leucine on glycogen synthesis.** Average newly synthesized glycogen expressed relative to the baseline (no stimulation) in the three biological replicates. Error bars denote standard deviations. Significant effects (p-value < 0.05) are highlighted with a star (*).

Results from the experiment carried out to measure glycogen synthesis are shown in Figure 4: leucine alone has no effect on glycogen synthesis, whereas both insulin and leucine + insulin conditions resulted in a significant increase with respect to the baseline (p-value = 0.0157 and 0.006, respectively), and the difference between the two conditions is statistically significant (p value =0.008). This finding is consistent with the observation that GSK3β phosphorylation was not altered by leucine and was increased instead both with insulin and leucine + insulin stimuli, given that GSK3β has an inhibitory role on glycogen synthesis until it is phosphorylated and the inhibition is released.

**Figure 5:**
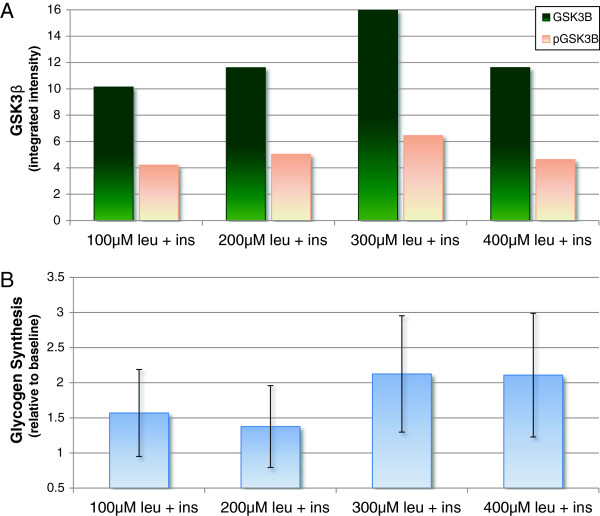
**Dose effect of leucine on GSK3β and glycogen synthesis. Panel A**: amount of GSK3β total (green) and phosphorylated (pink). **Panel B**: glycogen synthesis with varying concentrations of leucine.

As regards the effect of increasing leucine concentration on GSK3β and glycogen synthesis, it appears that 100 μM leucine concentration is able to elicit an elevated response, not far from those observed with 200-300-400 μM for both phosphorylated GSK3β (Figure 5, panel A) and glycogen synthesis (Figure 5, panel B). Even if data may suggest that the maximum effect is not reached with 100 μM, the low number of replicates do not allow to assess any statistically significant difference of pGSK3β and glycogen synthesis at higher concentrations.

## Discussion

The main finding of this work is that leucine modulates the phosphorylation of insulin signaling proteins in a way that is specific for each studied target, suggesting a coordinated regulation of insulin and leucine on insulin signaling. In particular, leucine is shown to increase the magnitude of insulin-mediated AKT phosphorylation at Ser473, which remained moderately elevated through 60 minutes with insulin, highly elevated with leucine + insulin, not altered with leucine alone. GSK3β, which is known to be phosphorylated by AKT, shows a similar pattern of activation. 100 μM of leucine are sufficient to increase the effect of insulin on GSK3β phosphorylation, which did not appear to be further upregulated at a significant extent if the concentration of leucine is increased above this value, although a positive trend can be observed from the data. Consistently, glycogen synthesis followed the same pattern of GSK3β, with leucine increasing the insulin-mediated glycogen synthesis by 50% (p-value 0.008). Leucine was also shown to increase the magnitude of insulin-mediated ERK phosphorylation; however, differently from pAKT and pGSK3β when treated by leucine + insulin, pERK shows a transient behavior, with a peak at 10′ followed by a return to the baseline condition. This behavior has already been reported in the literature under insulin stimulus and under epidermal growth factor (EGF) stimulus [14]. A transient ERK response might be obtained by a feedback of SOS, the son of sevenless homolog protein, which prevents a sustained activation of ERK that would result in continual cell proliferation [14].

mTOR, which is expected to be an additional target of leucine, showed partial inconsistency among different replicates. Lack of a clear effect by leucine at this site may suggest that leucine activates mTOR in human skeletal muscle by involving additional phosphorylation sites. The site investigated here is known to be phosphorylated by AKT; nonetheless, in a recent work [15], it was demonstrated that mTOR signaling complex 1 (mTORC1), which regulates p70S6K, contains mTOR phosphorylated predominantly on S2448 (the site monitored here), whereas mTOR signaling complex 2 (mTORC2), which regulates Akt through S473 phosphorylation, contains mTOR phosphorylated predominantly on S2481. This observation suggests to monitor in future studies mTOR complex formation rather than the targeted phosphorylation site used here.

In this experiment, only one replicate is available for phosphorylated p706SK at T389 site. pp706SK resulted to be 2-fold up-regulated under insulin and 18-fold up-regulated under leucine + insulin stimulus with a peak at 10′, and showed a significant effect of leucine when incubated before insulin stimulus. Our results are in agreement with [16], in which healthy adults were infused with leucine alone, insulin alone, or both leucine and insulin for 2 hours, and p70S6k and AKT phosphorylation were measured in vastus lateralis muscles showing upregulation under leucine + insulin stimulus. Analogously, in [17], phosphorylation of mTOR and p70S6K was transiently increased in human myotubes following leucine, insulin and leucine + insulin treatment. While results in [16,17] suggest that leucine and insulin act in an additive manner to increase p70S6k phosphorylation, other studies on skeletal muscle in pigs using an amino acid/insulin clamp [18], do not evidence any effect of amino acids on insulin induced stimulation of either IRS1 or Akt phosphorylation. As pointed out in the Introduction, in different studies a downregulation of leucine on insulin signaling was reported [7,8]; in particular, in [8] leucine deprivation in human HepG2 cells and mouse primary hepatocytes has been shown to improve hepatic insulin sensitivity by decreasing mammalian target of rapamycin/S6K1 signaling [8]. There might be different possible reasons of these controversial results in the literature among studies targeting leucine effect on insulin signaling. First, literature studies differ for being applied in vitro or in vivo, where a number of events involving different tissues and pathways can interact in an integrated fashion. Second, different studies are applied to different cell lines and tissues, in human or animal models. Third, experimental conditions are often different, being e.g. characterized by different amino acids and nutrient availability and/or different leucine incubation times/doses. For example, the deprivation of all amino acids in our experiment is likely to cause protein synthesis repression. Accordingly to [8], this leads to the activation of GCN2 and repression of p706SK, which was evident in our data since pp706SK at time 0′ was not detectable in our study in two out of three replicates. In our study, however, differently from [8], when cells are stimulated with leucine, the signaling system seems to respond to insulin in an enhanced way. It must be pointed out here that in our study muscle cells are in a state of amino acids deprivation and leucine availability, whereas in [8] cells, which are hepatic cells, are cultured with all amino acids but leucine. In this regards, since a wide range of signals, including nutrients, energy levels, growth factors, and amino acids are known to affect insulin signaling pathway, it is likely that experimental results strongly depend on the availability of combinations of the above variables. Moreover, the proteins involved in the insulin signaling pathway are known to exhibit a number of different phosphorylation sites, able to interact in different ways with different molecules, a complex set of regulatory mechanisms which are not completely understood, as for example in the case of mTOR [15]. Another reason of the discrepancies observed in the literature regarding leucine effect on insulin signaling pathway is that in most studies a single time point is measured and, as made evident by Figure 3, this can lead to different conclusions.

In summary, well controlled and standardized protocols, together with methods for monitoring phosphorylation events in a more high-throughput fashion, in experiments addressing the combined dynamic effect of insulin, leucine and other amino acids, in a variety of tissues and cell types, are needed by the scientific community to better clarify the complex regulatory pathway of insulin signaling system.

The present work, although not conclusive in terms of possible effects of leucine in insulin signaling pathway, demonstrates the importance of tracking phosphorylation events in time and with a proper sampling grid. Since phosphorylation events are likely to be transient, testing a single time point can lead to false negative findings due to lack of detection, even though phosphorylation occurs. Of note, these experiments only report on skeletal muscle cell lines. Although by using cell lines the environment studied cannot reproduce all of the complexities that occur in vivo, an advantage is that cell culture models are particularly suited to studying phosphorylation transient events because the timing of events can be studied with precision. It would be helpful to follow up with similar studies on human hepatic cell lines to determine whether a similar trend is observed in another cell type that is important for glucose metabolism.

## Conclusions

In order to examine the effect of leucine, an essential branched-chain amino acid, on muscle insulin signaling, a set of experiments was carried out to quantitate phosphorylation events along the insulin signaling pathway in human skeletal muscle cell cultures. Cells were serum starved overnight and exposed to insulin, leucine, or both, and phosphorylation events of key insulin signaling molecules were tracked over time so as to monitor time-related responses that characterize the signaling events and could be missed by a single sampling strategy limited to pre/post stimulus events.

Data show that leucine alone has no effect on AKT and GSK3β phosphorylation; however, leucine preincubation enhances the effect of insulin, amplifying the phosphorylation of AKT and GSK3β. This suggests that leucine does not directly promote AKT and GSK3β phosphorylation, but affects other signaling molecules that in turn, regulate AKT and GSK3β. The increase in GSK3β phosphorylation is then shown to lead to improved glycogen synthesis, being of potential interest for insulin-resistant states.

Leucine is also shown to increase the magnitude of insulin-mediated ERK phosphorylation; however, differently from AKT and GSK3β, ERK shows a transient behavior, with an early peak response, followed by a return to the baseline condition.

## Methods

### Experimental design

Muscle cells were deprived of serum overnight prior to exposure to three different stimuli: A) cells were stimulated with insulin at time 0′; B) cells were stimulated with leucine at time 0′; C) cells were pre-incubated with leucine for one hour and then stimulated with insulin (Figure 2). Cells were exposed to stimuli for the following durations: 0′, 2′, 5′, 10′, 30′, and 60′ and AKT, pAKT-S473, ERK1/2, ppERK1/2-T202-Y204, GSK3β, ppGSK3β-S21-S9, FOXO1a, pFOXO1-S256, mTOR, pmTOR-S2448, 4EBP1, pp4EBP1-T37-T46, P70S6K, and pP70S6K-T389 were measured using Western blots. Each experiment was repeated three times including cell cultures, so to have three available biological replicates. Measurements of each biological replicate were repeated in a number of technical replicates ranging from 2 to 5, performed on different blots. Glycogen synthesis at 90 minutes was also monitored in the three experimental conditions. All the experiments were repeated three times, thus three biological replicates were available for each condition.

In addition, the experiment with leucine + insulin stimulus was run with four different concentration of leucine: 100, 200, 300 and 400 μM; glycogen synthesis and ppGSK3α/β-S21-S9 at 90 minutes were monitored at these different concentrations.

### Cell cultures

Human skeletal muscle cells (SkMCs) and growth medium (SkGM) were purchased from Lonza. Cells were proliferated on 6-well plates between 5 and 7 passages at 37°C, 5% CO_2_, grown to 90% confluence and exposed to differentiation medium (DMEM:F12 w/2% Horse Serum and gentamycin) for ten days. Glucose was not added to the medium, but was present in the DMEM at 1000 mg/L. Cells were serum-starved with DMEM O/N and then switched to Earle’s Balanced Salt Solution (EBSS) for one hour, after which they were exposed to one of three media: 1) EBSS + 100 nM insulin, 2) EBSS + 400 μM leucine, or 3) EBSS + 400 μM leucine (1H priming) followed by EBSS + 100 nM insulin + 400 μM leucine. The 100 nM concentration represents a well-accepted level of insulin stimulation in cell cultures commonly found in the literature [8].

### Immunoblotting

Cells were lysed with Cell Signaling lysis buffer, sonicated, and centrifuged at 14KG for 30 minutes at 4°C. Cell Signaling Lysis buffer was composed by 20 mM Tris–HCl (pH 7.5), 150 mM NaCl, 1 mM Na2EDTA, 1 mM EGTA, 1% Triton, 2.5 mM sodium pyrophosphate, 1 mM b-glycerophosphate, 1 mM Na3VO4, 1 μg/ml leupeptin, We also added Halt Protease and Phosphatase inhibitor cocktail. The Pierce 660 reagent was used to determine protein concentrations and lysates were loaded onto Invitrogen Nupage gels for Western blot transfer using the Biorad semi-dry Trans-blot apparatus. Blots were blocked with Licor blocking buffer and incubated overnight at 4°C with target antibodies. *Cell Signaling* supplied all primary antibodies with the exception of FOXO1a and GSK3β antibodies, which were from *Abcam*. In details, the antibodies used were: Akt total – Cell Signaling #2920; Akt Phospho (Ser473) – Cell Signaling #9271; GSK-3B total – Abcam #ab2602; GSK-3B-phospho (Ser9) – Cell Signaling #9336; ERK1/2 total – Cell Signaling #4695; ppERK1/2 (T202-Y204) – Cell Signaling #9106; FOXO1a total – Abcam # ab89433; pFOXO1a (S256) – Cell Signaling #9461; mTOR – Cell Signaling #2983; pmTOR (S2448) – Cell Signaling #2971; 4EBP1 – Cell Signaling #9644; pp4EBP1 (T37-T46) – Cell Signaling #9459; P70S6K – Cell Signaling #9202; pP70S6K (T389) – Cell Signaling #9206. All were used at a 1:1000 concentration.

Band densities were quantified using the Licor Odyssey system using the integrated intensity value for each band. In the case protein concentration at time 0′ was difficult to detect because too low, it was necessary to concentrate lysates before loading them onto gels, using Vivaspin MWCO 30 kDa concentrating filters.

To allow for comparison between the total and the phosphorylated fraction of each protein, the time 0′ baseline sample was loaded on every gel and all other band densities were expressed relative to the baseline band to correct for the antibody efficiency. Moreover, to allow for comparison among different membranes, the ratios between phosphorylated and total proteins were calculated. Since for each protein, the total and the phosphorylated fractions were loaded in the same lane, this latter step also corrected for possible differences in the quantity of sample loaded in each gel. Therefore Beta-actin, which was also measured in each lane and is reasonably stable across the different lanes and blots, with mean equal to 16 and standard deviation equal to 3, was not used to normalize the data. Finally, technical replicates were averaged.

### Glycogen synthesis assay

Priming media were removed and media containing uniformly labelled 14C-glucose were added to the cultures. After 90 minutes, medium was removed, cells were washed with PBS and extracted with 1 ml 0.03% SDS (0.15 ml used for protein determination and 0.85 ml for glycogen extraction). Carrier glycogen was added, samples were heated to 95°C for 30 minutes and glycogen pellets were collected by centrifugation, washed with 70% ethanol, and resuspended in 200 μl distilled water. The incorporation of 14C-labeled glucose into glycogen was determined by 15 minute counts in a beta counter, using 10 ml optifluor liquid scintillation cocktail per sample. The stock 14C-glucose had a specific radioactivity of 7400 kBq/ml. The concentration used in the glycogen synthesis assay was 18 kBq/ml. Media was made using 31.63ul of the stock 14C-glucose into a total volume of 13 ml of media. Glycogen synthesis was expressed relative to its baseline. Each condition was run at least in triplicate.

### Statistical methods

The Standard t-test was applied to assess if, for each time point, the ratio between phosphorylated and total protein was significantly different from 1, i.e. the baseline value at time t = 0, or if there were differences between different treatments. P-value < 0.05 was considered significant.

In addition to that, the “Bounded Area” method, originally described in [13], was applied to the time courses of the single replicates, to identify, for each protein, the replicates whose overall profile is significantly modulated by insulin, leucine and/or leucine + insulin treatment. Briefly, the area bounded by the protein profile with respect to its baseline value was evaluated, and the protein was considered differentially expressed if this area exceeded a threshold *θ. θ* is the confidence threshold determined in correspondence to a significance level α, based on the null hypothesis distribution. This latter was derived by repeatedly sampling data from an error distribution, empirically obtained from the available biological replicates as explained in [19]. As already shown, the method is quite robust to random oscillation since the entire expression profile is considered, thus diminishing both false positive and false negative rates with respect to standard tests when few replicates are available. Moreover, both the precision and the recall of the test are enhanced by the fact that the entire expression profile is tested, rather than single time points [13,20]. In order to account for multiple testing, the significance level α was corrected for multiple testing so to control the global False Discovery Rate at 5% [21]. The analysis was applied separately for the three available biological replicates, thus allowing to check for consistency of different experiments. The “Bounded Area” method was applied also to test differences between different treatments.

Differences in glycogen synthesis and GSK3β measured at different doses of leucine incubation followed by insulin stimulus were assessed using standard ANOVA and t-tests. P-value < 0.05 was considered significant.

## Competing interest

The authors declare that they have no competing interest.

## Authors’ contributions

BDC participated in the design of the study, performed the statistical analysis and drafted the manuscript. FE helped to perform the statistical analysis and to draft the manuscript. SKN conceived the study and helped to draft the manuscript. AA participated in the design of the study and helped to draft the manuscript. GT participated in the study design and coordination and helped to draft the manuscript. All authors read and approved the final manuscript.
